# Estimation of the Real Incidence of a Contagious Disease Through a Bayesian Multilevel Model: Study of COVID-19 in Spanish Provinces

**DOI:** 10.3390/healthcare12222308

**Published:** 2024-11-19

**Authors:** David Hervás, Patricia Carracedo

**Affiliations:** Department of Applied Statistics, Operations Research and Quality, Plaza Ferrándiz y Carbonell, Universitat Politècnica de València, 03801 Alcoy, Spain; daherma@eio.upv.es

**Keywords:** contagious disease, Bayesian modeling, incidence prediction, COVID-19, Spain

## Abstract

Background: Pandemic outbreaks have emerged as a significant global threat, with the potential to cause waves of infections that challenge public health systems and disrupt societal norms. Understanding the underlying behavior of disease transmission can be of great use in the design of informed and timely public health policies. It is very common for many contagious diseases not to have actual incidence but rather incidence in a given subgroup. For example, in Spain, as of 28 March 2022, the incidence of COVID-19 in people under 60 years of age is not registered. Methods: This work provides a Bayesian methodology to model the incidence of any infectious disease in the general population when its cases are only registered in a specific subgroup of that population. The case study used was the coronavirus disease (COVID-19), with data for 52 Spanish provinces during the period of 1 January 2020 to 29 August 2022. Results: Explicitly, two multilevel models were proposed, one for people over or of 60 years of age and the other for people under 60 years of age. Performance of the models was 5.9% and 12.7% MAPE, respectively. Conclusions: Despite the limitations of the data and the complexity and uncertainty in the propagation of COVID-19, the models were able to fit the data well and predict incidence with very low MAPE.

## 1. Introduction

People continue to be weakened and die from infectious diseases due to the emergence of new disease-causing pathogens and the reemergence or evolution of old pathogens [1]. In recent years, more detailed monitoring of these diseases has been made possible by improved informatics, electronic data management, the ability to share and deposit data on the internet, rapid diagnostic tests and genetic sequence analysis [2]. These data can be modeled to assist in the design of practical disease control strategies and play a key role in the public health strategies of many countries. Statistical models are ideal as forecasting tools, and therefore, they can help us understand the behavior of the disease and thus be able to prevent its future spread waves [3]. COVID-19 is an infectious disease caused by the SARS-CoV-2 virus. In addition to being the greatest global public health threat of the century, COVID-19 has all the characteristics that allow it to be considered not just a viral pandemic, but a “systemic pandemic of health inequality” according to class, gender, age, ethnicity, migration status and place of residence [4,5]. Although the first outbreak of COVID-19 was detected in China in December 2019, it quickly spread to other countries. In particular, the first wave of infections hit Europe hard, especially Italy and Spain. A few weeks later, the United States was affected [6]. In particular, Spain was hit hard by this health crisis. At the start of 2023, more than 13 million positive cases and more than 100,000 deaths had been reported [7]. These figures represent 2.04% of positive cases and 1.73% of deaths compared to the total number of cases in the world. Due to its rapid spread, this virus caused a global pandemic state. Recent studies such as the one by Gutiérrez et al. [8] confirm the uneven distribution of COVID-19 cases between the different autonomous communities or regions of Spain. This rapid spread was mainly related to two factors: the high infection rate of the virus and the global mobility of humans [9].

In the face of the global epidemic caused by COVID-19, governments had to implement non-pharmaceutical interventions (NPIs) which are the only option available to delay and moderate the spread of the virus in a population. Some examples of NPIs are border closure, lockdowns, and social distancing measures, among others. All government actions have associated economic and social consequences. For this reason, it is of vital interest to make sound and accurate decisions about NPIs, since they will prevent a resurgence of COVID-19 or similar diseases [10].

Statistics is a good tool to identify intrinsic characteristics and dynamics of COVID-19 outbreaks [11]. Due to the availability of data from during the global pandemic, several statistical models have been implemented and validated to predict the behavior of the virus. For example, Orea and Álvarez [6] study the propagation of COVID-19 in the Spanish provinces and evaluate the effectiveness of the lockdown of the population through a standard spatial econometric model. The work of López-Mendoza et al. [12] analyzes the presence of convergence in the cumulative incidence of 14 days in Spanish provinces. The Phillips–Sul methodology was used to study the clustering of behaviors between provinces, and an ordered logit model was estimated to understand the forces that drive the creation of the different convergence clubs. Paez et al. [13] modeled the incidence of reported COVID-19 cases per 100,000 inhabitants as an inter-regional contagion process using apparently unrelated (SUR) spatial regressions including temperature, humidity, sunshine, GDP per capita, percentage of older adults in the population, population density, and the presence of mass transit systems. Aràndiga et al. [14] present a model for the simulation of COVID-19 in various areas of Spain using mobility data. This model, based on the less common compartmental model called SAIR (Suspected–Asymptomatic–Infected–Retired), is able to qualitatively simulate the spread tendencies of small outbreaks. Jalilian and Mateu [15] use a spatio-temporal hierarchical Bayesian model to explain the temporal and spatial variations in the daily number of new confirmed cases in Spain, Italy and Germany. The common link of all these studies is to predict the incidence of COVID-19 in Spain. However, unlike them, we deal with the issue of forecasting incidence in the whole population with the short-term historical incidence of a specific subgroup.

Regarding COVID-19 databases, there are many national and international organizations that have published open data on the number of cases and deaths [15,16]. However, these data often suffer from incompleteness and inaccuracy, which are important limitations for any study of COVID-19 [17]. It is important to note that, in the case of Spain, as of 28 March 2022, the incidence of COVID-19 in persons under 60 years of age was not recorded.

In view of the above, the aim of this work is twofold. First, we develop and implement a statistical methodology to predict and explain the short-term behavior of any infectious disease. As a case study, the incidence of COVID-19 in provinces in Spain was selected. Second, we use this to predict the true incidence of COVID-19 for the total population, including people under 60 years of age. It is very common for many contagious diseases not to have actual incidence, but rather incidence in a given subgroup. This always implies a misestimation of the incidence [18]. For this reason, the method we propose to predict the incidence in people under 60 years of age as the ratio between incidence in the general population and incidence in the subgroup is interesting. In this way, we would always obtain non-underestimated estimates of incidence. Thus, the added value of our model with respect to others is that our model does not need previous incidence data from the whole population to predict the future incidence in that population. This is of vital importance since complete data for the entire population may not be available for a specific infectious disease, reducing the need to collect large-scale data while still allowing for accurate and timely forecasts. Predicting behavior in specific subgroups of the population is essential for better understanding the dynamics of infectious diseases, optimizing resources, and developing more effective interventions. To address this, a Bayesian methodology is used because it offers key advantages over frequentist models, including the ability to incorporate prior information, update parameter estimates with new data, and provide a full probability distribution to better capture uncertainty. They are also more flexible in handling complex models and missing data [19].

The structure of the paper is as follows. Section 2 starts by describing the open data resources used in this study. This section ends with a detailed exposition of the proposed Bayesian multilevel methodology. Section 3 presents the main results of the exploratory analysis of the database and the application of the statistical methodology, and finally, in Section 4, the most important conclusions of the study together with some lines of future research are shown.

## 2. Data and Methods

### 2.1. Data Description

The data were downloaded from the Carlos III Health Institute webpage https://cnecovid.isciii.es/covid19/ (accessed on 4 December 2023). This study takes into account temporal evolution of 58,708 records of COVID-19 detected in 52 Spanish provinces during the period of 1 January 2020 to 29 August 2022. As mentioned before, currently in Spain, only data for people over or of 60 years of age were being reported. For this reason, for persons over or of 60 years of age, data are available until 29 August 2022. While, for persons under 60 years of age, data are until 27 March 2022. Finally, it should be noted that the database contains the following variables: province, sex, age group, date and number of cases.

Statistical analysis was performed using the free software R (Version 4.4.1) [20] and some specific R-packages: clickR [21], brms [22], ggplot2 [23], mapspain [24], sf [25].

### 2.2. Data Analysis Methods

We first debugged the database, correcting some sources of noise. For example, the acronym of the province called Navarra is NA which implies conflicts with missing values. Once these mistakes were corrected, we eliminated 10,161 cases where the provinces were missing but had events. The variable that is modeled in this study is the number of cases per 100,000 people. For its calculation, the reference population was obtained from the Statistics National Institute (https://www.ine.es/ (accessed on 4 December 2023)).

Statistical modeling of the evolution in number of cases per 100,000 people was performed using Bayesian methodology. For people over or of 60 years of age, the Bayesian linear model with a natural cubic spline for the day by province was applied according to Equation (1):

(1) $$\sqrt{cases\_100000p>60}∼\beta_{0}+f_{1}\left(day\right)*\beta_{i}province+\varepsilon$$

where $f_{1}\left(day\right)$ defines a n∗5 (degrees of freedom) basis matrix of piecewise-cubic splines enforced to be linear beyond the boundary knots, and $\beta_{i}$ stands for the coefficients of each province. The model included an interaction to account for differential effects of the variable *day* on each different province. It should be noted that this model took the last 20 days to predict the next 7 days. Weak regularizing priors were defined for the coefficients of the model ($\beta_{i}$) using normal distribution with mean = 0 and standard deviation = 3. The prior distributions for the coefficients of the basis matrix of piecewise-cubic splines were flat.

From modeling the total population, we obtained the prediction for people under 60 years of age following Equation (2).

(2) $$log\left(\frac{cases\_100000p<60}{cases\_100000p>60}\right)∼\beta_{0}+\beta_{i}province+\varepsilon$$

Priors for $\beta_{0}$ and $\beta_{i}$ were the same as in Equation (1). Once the ratio was obtained, the posterior distribution of cases for people under 60 years was obtained by calculating the Kronecker product of the posteriors obtained in Equations (1) and (2).

(3) $$F_{x}\left(cases\_100000p<60\right)=F_{x}\left(cases\_100000p>60\right)⊗F_{x}\left(\frac{cases\_100000p<60}{cases\_100000p>60}\right)$$

The performance of the model was measured with mean absolute percentage error (MAPE).

## 3. Results

In this section, we describe the main results of our analysis. We first present a general descriptive analysis of the data and, then, the modeling results for people over and under 60 years of age.

### 3.1. Descriptive Analysis

**Figure 1:**
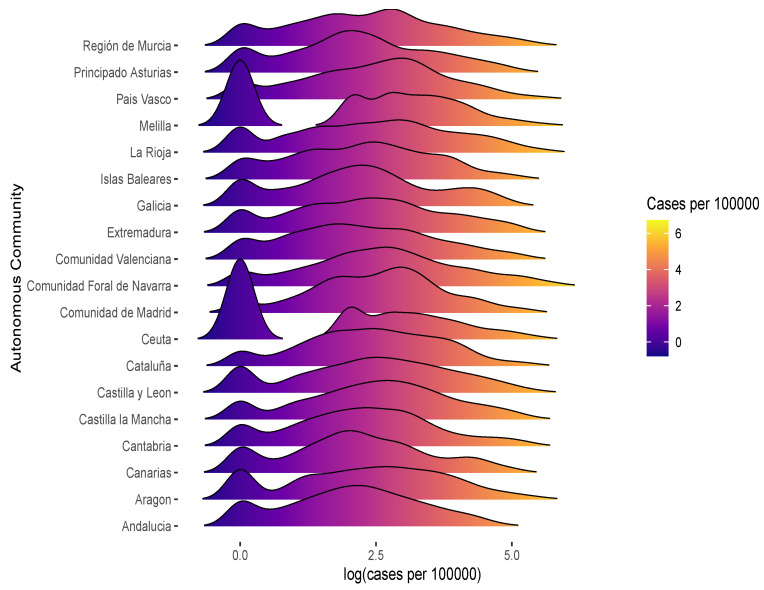
Accumulated number of COVID-19 cases by Spanish province.

In this work, 58,708 outbreaks in 52 Spanish provinces between 1 January 2020 and 29 August 2022 were analyzed. Figure 1 represents the distribution of the logarithm of the number of cases per 100,000 people for each autonomous community in a stacked density line plot. In this case, for the sake of brevity, the 52 provinces were grouped into 19 autonomous communities. The representation was carried out by superimposing several frequency densities of the numeric variable for autonomous communities, which created a ridges effect. According to the legend, blue indicates the lowest number of cases and yellow the highest. It can be seen that the communities with the fewest cases of COVID-19 were Ceuta and Melilla. On the other hand, the communities with the highest incidence of the virus were Madrid and Cataluña. These results are in line with works such as Gutiérrez et al. [8], Ponce-de Leon et al. [9]

### 3.2. Statistical Modelling

This section shows and details the results for each model.

#### 3.2.1. People over or Equal to 60 Years of Age

**Figure 2:**
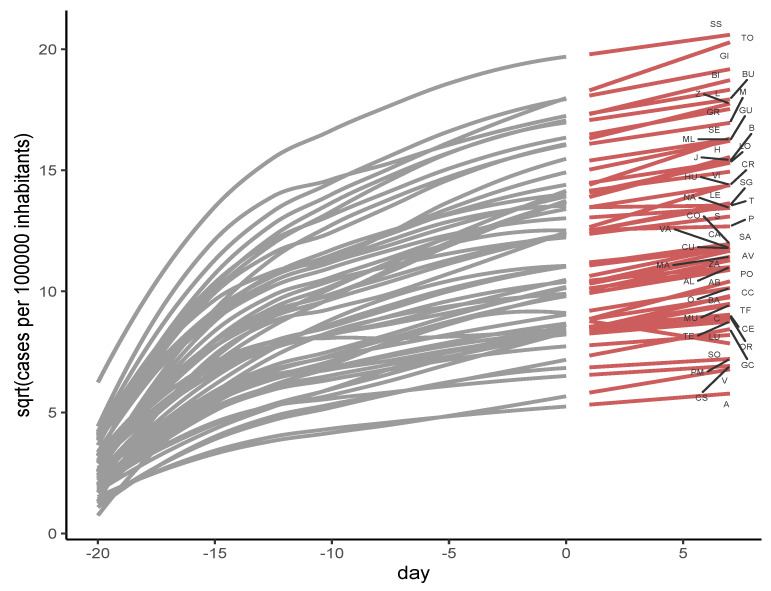
Prediction of the number of cases per 100,000 for people over or of 60 years of age.

Finally, we model the evolution of the number of cases per 100,000 for people over or of 60 years of age using a Bayesian methodology. Specifically, a Bayesian linear model with a natural cubic spline for the day by province was used (Equation (1)). Figure 2 represents the prediction for the number of cases per 100,000 for people over or of 60 years, from day −20 to day 7 of the analysis based on the data from the previous 20 days (see AbbreviatIons Section). It is worth noting that the convergence measure for this model Rhat had a value of 1. Rhat value, also known as the Gelman shrink factor and the potential scale reduction factor, shows the convergence of the algorithm [26]. The model is deemed convergent if the Rhat value is equal to 1 [22].

Next, Table 1 represents the observed value, prediction, and 95% credible interval of the model for individuals over or of 60 years of age. The prediction has been made for 5 days into the future from any given day for the two autonomous communities with the highest number of cases for COVID-19 (Madrid and Cataluña) and the two autonomous cities with the lowest number of cases (Ceuta and Melilla). It should be noted that the MAPE is around 5.9%.

#### 3.2.2. People Under 60 Years of Age

**Figure 3:**
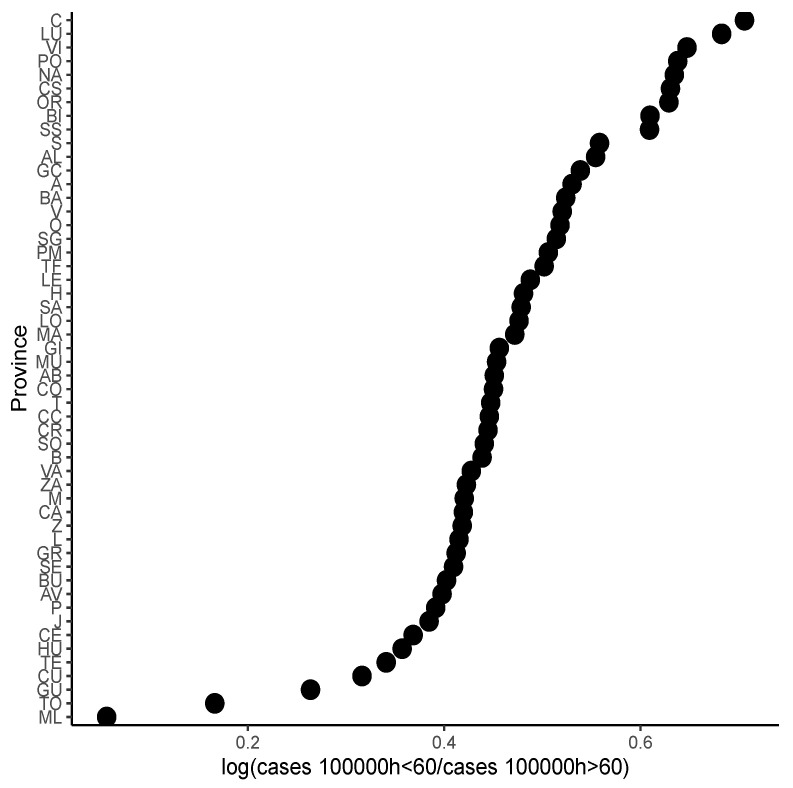
Prediction of the ratio by province.

In the same way, Bayesian methodology was used to model the cases per 100,000 for people under 60 years. In this case, first, the logarithm of ratio of people over or of 60 years of age and people under 60 years of age was obtained as the predictor variable in the model. In this case, the ratio was modeled with a Bayesian linear model by province (Equation (2)). Then, the posterior distribution of cases for people under 60 years was obtained by making the Kronecker product as in Equation (1). Figure 3 represents the prediction of ratio of the last day of the analysis using information from the previous 20 days for each province (see Abbreviations Section). Just as before, the Rhat value was 1, indicating that the model had converged correctly.

Next, Table 2 represents the observed value, prediction, and 95% credible interval of the model for individuals under 60 years old. It should be recalled that the ratio of the number of cases was used here to make the prediction (see Equation (3)). In the same way as Section 3.2.1, the prediction was carried out for 5 days after the current one in the two communities with the highest and lowest incidence of COVID-19. In this case, the MAPE is around 12.7%.

## 4. Discussion and Conclusions

The COVID-19 pandemic has led to a focus on mathematical modeling with the aim of assisting both governments and public institutions in their decision-making. On the one hand, there are people living with another disease who may have an increased risk of COVID-19 infection or severity [27]. Also, there are people living with another disease who may have an increased risk of COVID-19 infection or severity. On the other hand, the capacity of healthcare systems is diminished by the new challenges and resource adaptations needed to combat COVID-19 [28]. But it is not only COVID-19 that has caused global pandemics. It is worth noting that, other neglected pandemics caused by communicable diseases such as HIV/AIDS, tuberculosis, and malaria must not be forgotten [29]. According to the World Health Organization [30], in 2019, these three diseases along with viral hepatitis (also counting cirrhosis and liver cancer secondary to hepatitis) were among the 20 leading causes of death worldwide. Thus, the United Nations 2030 Agenda for Sustainable Development, in its goal 3.3, focuses on rooting out these infectious diseases [31].

Following our aim to show a statistical methodology for modeling any contagious disease, we selected COVID-19 as a case study. This disease was selected for two main reasons. The first is to have access to more data, and the second is because, in a relatively short period of time since its onset, COVID-19 caused significantly more deaths compared to malaria, hepatitis and HIV/AIDS [32]. The dataset used contains all COVID-19 cases reported in Spain by province during the period of 1 January 2020 to 29 August 2022 for people over or of 60 years of age and until 27 March 2022 for persons under 60 years of age. It is worth mentioning that there are some limitations in the database, such as incomplete and inaccurate data, unavailability or inaccuracy of relevant variables, as well as the unknown nature of the new COVID-19 virus [15].

However, it is important to correctly model infectious diseases in order to detect patterns of virus behavior and prevent their spread. This can help both governments and public health agencies to design public policies in future waves of pandemics caused by any infectious disease.

Infectious diseases were modeled from a multilevel Bayesian model. Specifically, COVID-19 was used as a case study. For people over 60 years of age, the Bayesian linear model with a natural cubic spline for the day by province was applied.

As COVID-19 data are currently recorded in Spain only for people over 60 years of age, it is also important to estimate the real incidence in the whole population. For people over 60 years of age, a Bayesian linear model with a natural cubic spline for the day by province was used. The added value of this work lies in its use for people over 60 years of age. First, the logarithm of ratio of people over 60 years of age and people under 60 years of age was modeled with a Bayesian linear model by province. We would like to highlight that, unlike other models that make predictions for the population based on the behavior of past incidence data from that same population, the model proposed in this work does not require previous incidence data from the entire population; it only needs incidence data from one subgroup of the population and an estimate of the incidence ratio between groups. This fact is very important because, regarding COVID-19, for example, no data were collected in Spain for people under 60 years of age, who were also infected. As the main obstacle of this work, we would like to state that it is difficult to compare the results of this model with others, as they use different methodologies with different aims. In fact, the methodology proposed in this work for using the incidence of a subgroup to obtain estimates of future incidence in the whole population could easily be expanded to adapt other modeling approaches. The key is that, unlike other approaches, this method is able to make predictions for a specific age subgroup for which there are no previous incidence data. The rest of the works do not deal with this problem, so the predicted incidence in these cases cannot be extrapolated to that of the whole population. This procedure could be applied to any communicable disease for which data are available for only a subset of the population. Despite data limitations and the complexity and uncertainty of the spread of COVID-19, the models were able to fit the data well and make predictions with very low MAPE. We would like to point out that, although COVID-19 was used in this work, this methodology can be applied to any infectious disease.

However, it should be noted that a step forward would be to include information about covariates, temporal correlation and spatial dependence due to a neighborhood relation between regions in the modeling to improve the model’s predictive capabilities.

## Figures and Tables

**Table 1:** Real value, prediction, and credible interval of the model for individuals over or of 60 years of age.

	Observed Value	Predicted Value	95% Credible Interval
Madrid	293.90	279.55	[258.82; 325.06]
Cataluña	231.70	227.91	[208.05; 269.78]
Ceuta	72.48	77.81	[73.00; 105.96]
Melilla	280.37	254.00	[233.32; 301.12]

**Table 2:** Real value, prediction, and credible interval of the model for individuals under 60 years old.

	Observed Value	Predicted Value	95% Credible Interval
Madrid	592.24	611.30	[506.39; 2186.92]
Cataluña	449.78	481.17	[393.71; 1745.14]
Ceuta	83.41	103.74	[70.41; 511.17]
Melilla	490.32	487.56	[421.43; 1464.04]

## Data Availability

Data can be publicly accessed from the Carlos III Health Institute webpage https://cnecovid.isciii.es/covid19/ (accessed on 4 December 2023).

## Abbreviations

C: A Coruña, A: Alicante, AB: Albacete, AL: Almería, VI: Álava, O: Asturias, AV: Ávila, BA: Badajoz, B: Cataluña, BI: Vizcaya, BU: Burgos, CC: Cáceres, CA: Cádiz, S: Cantabria, CS: Castellón, CE: Ceuta, CR: Ciudad Real, CO: Córdoba, CU: Cuenca, SS: Guipúzcoa, GI: Girona, GR: Granada, GU: Guadalajara, H: Huelva, HU: Huesca, PM: Baleares, J: Jaén, LO: La Rioja, GC: Las Palmas, LE: León, L: Lleida, LU: Lugo, M: Madrid, MA: Málaga, ML: Melilla, MU: Murcia, NA: Navarra, OR: Ourense, P: Palencia, PO: Pontevedra, SA: Salamanca, TF: Santa Cruz de Tenerife, SG: Segovia, SE: Sevilla, SO: Soria, T: Tarragona, TE: Teruel, TO: Toledo, V: Valencia, VA: Valladolid, ZA: Zamora, Z: Zaragoza.
